# Changing trends and serotype distribution of *Shigella* species in Beijing from 1994 to 2010

**DOI:** 10.1186/1757-4749-5-21

**Published:** 2013-08-07

**Authors:** Yuanli Mao, Enbo Cui, Chunmei Bao, Zhenhong Liu, Suming Chen, Juling Zhang, Huan Wang, Chenglong Zhang, Jing Zou, John D Klena, Baoli Zhu, Fen Qu, Zhiyun Wang

**Affiliations:** 1 Clinical Diagnostic Center, 302nd Hospital of the People’s Liberation Army, Beijing 100039, PR China; 2 Institute of Microbiology, Chinese Academy of Sciences, Beijing 100101, PR China; 3 School of Basic Medical Sciences, Peking University, Beijing 100083, PR China

**Keywords:** Shigellosis, *Shigella*, Serotype, Beijing hospital

## Abstract

*Shigella* species are a common cause of acute diarrheal disease in China. In this study, we characterized the changing trends and serotype distribution of *Shigella* species in Beijing from 1994 to 2010. A total of 5999 *Shigella* strains were isolated and serotyped from the 302nd Hospital in Beijing. The annual number of *Shigella* isolates reached a peak (n = 1192; 19.84%) in 1996 and then decreased annually, reaching the lowest point (n = 24; 0.41%) in 2010. *S. flexneri* 2a and *S. sonnei* were the most frequently isolated *Shigella*, with their respective isolates making up 53.3% and 27.6% of the total. Isolates of *S. flexneri* 4c, 4a, and *x* made up 3% respectively of the total isolates. Significant decreases in percentage of *S. flexneri* over time were observed. *S. sonnei* surpassed *S. flexneri* 2a as the predominant serotype in 2000. Most isolates were recovered from July to September; 13.6% of the isolates were recovered from children aged 0 to 5 years, and 16% were recovered from those aged 21 to 25 years. *S. flexneri* 2a and 5 were recovered mostly from males (33.41%, *p* < 0.001; and 0.46%, *p* < 0.001%; respectively), whereas *S. flexneri* 2b and 6, and *S. sonnei* were most often isolated from females. Continuous monitoring of *Shigella* showed that all 4 species and 27 serotypes were present in Beijing, China, during the study period. The emergence of *S. sonnei* and the overall decreasing isolation rate of *Shigella* in Beijing can potentially aid in the development of vaccine and control strategies for shigellosis in the city.

## Introduction

*Shigella* species are a common cause of acute diarrheal disease worldwide, with an estimated 167 million cases per year and resulting in approximately 1.1 million deaths; 97.6% of the cases occur in developing countries [1]. According to the Chinese National Infectious Disease Internet Reporting System, the annual incidence of shigellosis in China made it rank in the top three of the most notable infectious diseases for four consecutive years (2005 to 2008), with close to 500000 cases of shigellosis per year (http://www.moh.gov.cn); this number is now widely believed to be underestimated [2].

Shigellosis is caused by four species, *S. dysenteriae*, *S. flexneri*, *S. boydii*, and *S. sonnei*. *Shigella* species can be identified by serotyping with group-specific antigens; serotyping is based on structural differences within the O-antigen repeating unit of lipopolysaccharide [3]. A total of 47 serotypes of *Shigella* have been recognized, including 15 for *S. flexneri*, 13 for *S. dysenteriae*, 18 for *S. boydii*, and a single one for *S. sonnei*[4]. The distribution of species and serotypes of *Shigella* is heterogeneous over time and place [5].

The World Health Organization has made the development of a safe and effective vaccine against *S. flexneri*[1,6-8], but the vaccine effectiveness depends on the distribution patterns of local species and serotypes, because only type-specific immunity has been demonstrated in humans [9-12] and moreover cross-serotype protection is controversial [11,13].

According to a previous multicenter study of *Shigella* diarrhea in six Asian countries, *S. flexneri* is the most common species in Bangladesh, China, Pakistan, Indonesia, and Vietnam; whereas *S. sonnei* is predominant in industrialized countries [14]. Two recent reports have indicated that *S. flexneri* 2a is the most frequently isolated *Shigella* organism in China [15,16]. However, these reports may not be generalizable for the whole China; the time period of these studies is short, and surveillance is performed only in less-developed areas of China.

Little is known about the distribution of *Shigella* serotypes in Beijing, the political, educational, cultural, and economic center of China with a population of over 30 million. The present study describes the trends in *Shigella* species and their serotypes isolated from patients with diarrhea in a national infectious disease hospital in Beijing, China, from 1994 to 2010.

## Materials and methods

### Study sites and settings

The location was a clinical diagnostic center at the 302nd Hospital of the People’s Liberation Army in Beijing, China. The 302nd Hospital is the largest infectious disease teaching hospital in Beijing, China, with 1300 beds and receiving more than 36400 patients annually. From January 1994 to December 2010, fresh stool specimens were collected from patients with diarrhea and clinically suspected dysentery. The specimens were submitted to the microbiology laboratory of the 302nd Hospital. All experimental research have been performed with the approval of ethics committee of 302nd Hospital of the People’s Liberation Army, with reference number 2004013D.

### Bacterial isolation

Samples were cultured for *Shigella* by streaking diarrheal stools directly onto *Salmonella*–*Shigella* agar (Tian Tan Biologic Technology Company, Beijing, China) and incubating for 24 h at 37°C. *Shigella*-like colonies were selected and subcultured on Kligler iron agar (Qingdao Hope Biol-Technology Co., Ltd., Shandong, China). Except for *S. flexneri* 6 and *S. boydii* 14, *Shigella* spp. produce an alkaline slant and an acid butt but do not produce gas or H_2_S. As a species, *Shigella* organisms are characteristically nonmotile and lack the enzyme lysine decarboxylase.

#### Serotyping

Serologic identification was performed by slide agglutination with polyvalent somatic (O) antigen grouping sera, followed by testing with available monovalent antisera for specific identification of serotypes according to the manufacturer’s instructions (Denka Seiken, Japan). Only one *Shigella* isolate per patient per diarrheal episode was included in the analysis.

#### Statistical analysis

Statistical comparisons were performed using the CHISS software (version 2001, Yuan YiTang Sci-Tech Co., Ltd., Beijing, China). Categorical data were expressed as percentages and calculated using a chi-square test, and *p* ≤ 0.05 was considered statistically significant.

## Results

From 1994 to 2010, a total of 5999 *Shigella* isolates were collected from 372 inpatients and 5627 outpatients with diarrhea. All patients acquired diarrheal infection in the community, most likely through the ingestion of contaminated food and water. Cases for which the diarrheal infection was acquired through travel or via sexual contact were excluded from the analysis. Among the 5999 *Shigella* isolates, 12 were of an unknown subgroup (i.e., either not reported, not further typed, or untypeable); a final total of 5987 isolates were further analyzed in this study.

### Subgroup trends

**Figure 1:**
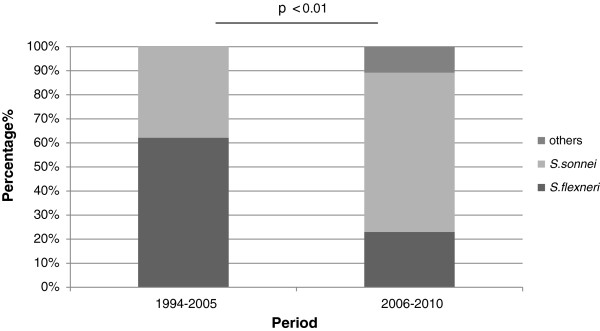
**The distribution of *Shigella *species in Beijing from 1994 to 2010.**

**Figure 2:**
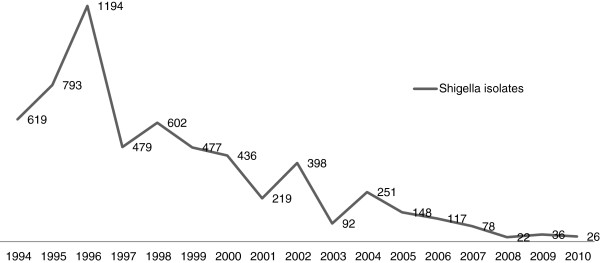
**Trends over time for *Shigella *spp. isolated from Beijing between 1994 and 2010.**

**Figure 3:**
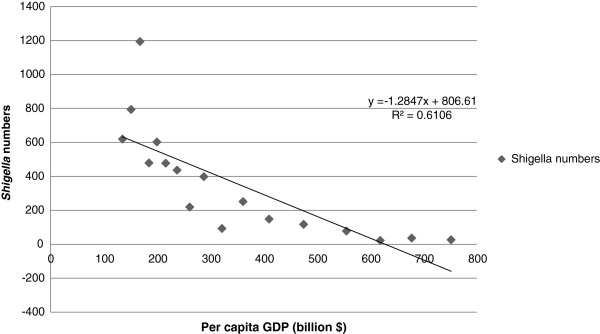
Number of isolates by per capita GDP (adjusted for purchasing power parity) of China from 1994 to 2010.

A statistically significant decreasing trend in *S. flexneri* and an increasing trend in *S. sonnei* were observed by chi-square analysis (*p* < 0.01) (Figure 1). The trends in *Shigella* spp. isolated from Beijing between 1994 and 2010 are shown in Figure 2. The recording of annual *Shigella* isolation began in 1994, and the maximum number of isolates was reported in 1996 (n = 1194). The annual total number of isolated *Shigella* organisms had been decreasing since then, reaching a low point in 2008 (n = 22). This trend may be related to the strict hygiene inspection and adequate sanitation during the 2008 Olympic season [17]. Four peaks were observed during the 17-year collection period. Peak 1 appeared in 1996, with subsequent peaks in 1998 (peak 2, n = 602), 2002 (peak 3, n = 398), and 2004 (peak 4, n = 251). A sudden decrease in *Shigella* isolation was observed more in 2003 than in 2002 and 2004; one possible explanation is that resources were redirected to identify severe acute respiratory syndrome cases in China in 2003, thereby limiting bacterial diarrheal isolation. It should be noted that as the numbers of observed cases of shigellosis were decreasing, China’s per capita gross domestic product (GDP) was increasing (Figure 3).

### Subgroups

**Figure 4:**
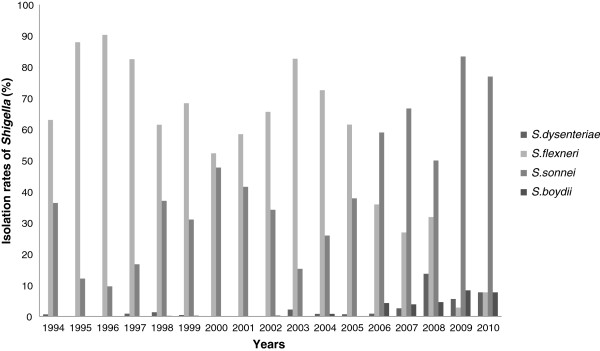
**Isolation rates of *Shigella *spp. by subgroup in Beijing from 1994 to 2010.**

The distribution of typeable *Shigella* during the study period was *S. flexneri,* 71.7% (n = 4295); *S. sonnei,* 27.3% (n = 1639); *S. dysenteriae,* 0.55% (n = 33); and *S. boydii,* 0.33% (n = 20). The distribution of *Shigella* species changed over the 17-year observation period (Figure 4). Between 1994 and 2005, *Shigella* isolation rates were largely driven by *S. flexneri*, reaching peak numbers in 1996 when 90% of all isolated *Shigella* were *S. flexneri*. In 2006, *S. sonnei* became the dominant subgroup. In 2009, the lowest percentage of isolated *S. flexneri* (6%) was recorded. The apparent isolation rates of *S. boydii* and *S. dysenteriae* increased during this period, e.g., *S. boydii* isolation rates increased from 0% to 4.2% (n = 5), 3.8% (n = 3), 4.5% (n = 1), 8.3% (n = 3), and 7.6% (n = 2) in 2006, 2007, 2008, 2009, and 2010, respectively. However, the absolute numbers of *S. boydii* and *S. dysenteriae* did not change during this period, remaining between 0 and 5 per year. This result suggests that although uncommon, sources of *S. dysenteriae* and *S. boydii* remain in the Beijing area.

### *Serotypes of* S. flexneri *and* S. sonnei

As revealed by 17 years’ worth of data, 27/47 (57.4%) *Shigella* serotypes were identified; these serotypes included *S. boydii* serotypes 1, 2, 5, 15, and 17; *S. dysenteriae* serotypes 1, 2, 3, 4, 5, 7, and 8; *S. flexneri* serotypes 1a, 1b, 2a, 2b, 3a, 3b, 4a, 4b, 4c, 5, 6, x, and y; and *S. sonnei*. Given the small numbers of *S. boydii* and *S. dysenteriae* (n = 53 combined), only isolates of *S. flexneri* and *S. sonnei* were further analyzed. Surveillance data indicated large differences in the serotypes of *S. flexneri* isolated between 1994 and 2010, in addition to the change in dominant species in 2006 (Table 1). *S. flexneri* 2a and *S. sonnei* were observed in every year of the study, and *S. sonnei* surpassed *S. flexneri* 2a as the predominant single *Shigella* type in 2000. The number and proportion of *S. flexneri* 2a isolates decreased from 42.6% (n = 262) in 1994 to 13.6% (n = 3) in 2010, whereas the proportion of *S. sonnei* increased from 36.6% (n = 225) to 82.3% (n = 20) during the entire period of data collection. *S. flexneri* 2a ranked the first among the *S. flexneri* subtypes except in 2003, 2004, and 2005. *S. flexneri* 4c, 4a, and *S. flexneri* x were seldom isolated, accounting for only about 3% (n = 180) of the 5934 *S. flexneri* and *S. sonnei* isolates. However, *S. flexneri* 4c was the most frequently isolated *S. flexneri* serotype in 2004 (26.4%, n = 65) and 2005 (20.3%, n = 30). *S. flexneri* 4c and *S. sonnei* were isolated in equal numbers in 2004.

**Table 1:** **Distribution of *S. flexneri *and *S. sonnei *serotypes in Beijing between 1994 and 2010**

	**No. (%) of isolates**																													
**Surveillance time**	**Total**		***S. flexneri *serotype 1a**		***S. flexneri *serotype 1b**		***S. flexneri *serotype 2a**		***S. flexneri *serotype 2b**		***S. flexneri *serotype 3a**		***S. flexneri *serotype 3b**		***S. flexneri *serotype 4a**		***S. flexneri *serotype 4b**		***S. flexneri *serotype 4c**		***S. flexneri *serotype 5**		***S. flexneri *serotype 6**		***S. flexneri *serotype x**		***S. flexneri *serotype y**		***S. sonnei***	
1994	615	(10.36)	113	(18.37)	0	(0.00)	262	(42.60)	3	(0.49)	0	(0.00)	0	(0.00)	3	(0.49)	3	(0.49)	2	(0.33)	1	(0.16)	2	(0.33)	0	(0.00)	1	(0.16)	225	(36.59)
1995	793	(13.36)	4	(0.50)	1	(0.13)	632	(79.69)	0	(0.00)	2	(0.25)	0	(0.00)	14	(1.77)	2	(0.25)	10	(1.26)	0	(0.00)	8	(1.01)	5	(0.63)	20	(2.52)	95	(11.98)
1996	1192	(20.09)	5	(0.42)	1	(0.08)	984	(82.55)	0	(0.00)	6	(0.50)	0	(0.00)	23	(1.93)	2	(0.17)	28	(2.35)	1	(0.08)	3	(0.25)	4	(0.34)	21	(1.76)	114	(9.56)
1997	474	(7.99)	2	(0.42)	0	(0.00)	365	(77.02)	0	(0.00)	1	(0.21)	0	(0.00)	4	(0.84)	1	(0.21)	6	(1.26)	1	(0.21)	0	(0.00)	6	(1.26)	9	(1.90)	79	(16.65)
1998	600	(10.11)	1	(0.17)	2	(0.33	340	(56.67)	0	(0.00)	0	(0.00)	0	(0.00)	0	(0.00)	3	(0.50)	4	(0.67)	0	(0.00)	3	(0.50)	8	(1.33)	11	(1.83)	228	(38.00)
1999	475	(8.00)	6	(1.26)	3	(0.63)	192	(40.42)	3	(0.63)	11	(2.32)	2	(0.42)	4	(0.84)	2	(0.42)	2	(0.42)	2	(0.42)	24	(5.05)	54	(11.37)	23	(4.84)	147	(30.95)
2000	433	(7.30)	7	(1.62)	1	(0.23)	132	(30.48)	19	(4.39)	7	(1.62)	0	(0.00)	0	(0.00)	2	(0.46)	3	(0.69)	23	(5.31)	8	(1.85)	16	(3.70)	7	(1.62)	208	(48.04)
2001	214	(3.61)	1	(0.47)	1	(0.47)	81	(37.85)	6	(2.80)	0	(0.00)	1	(0.47)	11	(5.14)	1	(0.47)	15	(7.01)	0	(0.00)	1	(0.47)	4	(1.87)	0	(0.00)	92	(42.99)
2002	397	(6.69)	8	(2.02)	0	(0.00)	116	(29.14)	57	(14.37)	0	(0.00)	0	(0.00)	13	(3.28)	3	(0.76)	11	(2.77)	1	(0.25)	1	(0.25)	43	(10.84)	8	(2.02)	136	(34.30)
2003	88	(1.48)	4	(4.55)	2	(2.27)	13	(14.77)	15	(17.05)	0	(0.00)	0	(0.00)	12	(13.64)	7	(7.95)	9	(10.23)	0	(0.00)	0	(0.00)	9	(10.23)	4	(4.55)	13	(14.77)
2004	247	(4.16)	4	(1.62)	1	(0.41)	7	(2.68)	16	(6.49)	3	(1.22)	0	(0.00)	58	(23.52)	8	(3.24)	65	(26.36)	1	(0.41)	0	(0.00)	17	(6.89)	2	(0.81)	65	(26.36)
2005	148	(2.49)	3	(2.03)	0	(0.00)	9	(5.96)	11	(7.44)	0	(0.00)	0	(0.00)	25	(16.91)	3	(2.03)	30	(20.30)	0	(0.00)	3	(2.03)	7	(4.74)	1	(0.68)	56	(37.89)
2006	112	(1.89)	7	(6.25)	1	(0.89)	11	(9.83)	6	(5.36)	0	(0.00)	0	(0.00)	5	(4.46)	7	(6.25)	0	(0.00)	0	(0.00)	0	(0.00)	4	(3.57)	2	(1.79)	69	(61.60)
2007	74	(1.25)	5	(6.77)	0	(0.00)	10	(13.40)	1	(1.35)	0	(0.00)	0	(0.00)	1	(1.35)	2	(2.71)	1	(1.35)	0	(0.00)	0	(0.00)	1	(1.35)	1	(1.35)	52	(70.36)
2008	17	(0.29)	1	(5.81)	0	(0.00)	2	(12.80)	1	(5.81)	0	(0.00)	0	(0.00)	0	(0.00)	1	(5.81)	2	(11.63)	0	(0.00)	0	(0.00)	0	(0.00)	0	(0.00)	10	(58.14)
2009	31	(0.52)	0	(0.00)	0	(0.00)	1	(3.54)	0	(0.00)	0	(0.00)	0	(0.00)	0	(0.00)	0	(0.00)	0	(0.00)	0	(0.00)	0	(0.00)	0	(0.00)	0	(0.00)	30	(96.46)
2010	24	(0.40)	0	(0.00)	1	(4.11)	3	(13.59)	0	(0.00)	0	(0.00)	0	(0.00)	0	(0.00)	0	(0.00)	0	(0.00)	0	(0.00)	0	(0.00)	0	(0.00)	0	(0.00)	20	(82.30)
Total	5,934	(100)	171	(2.88)	14	(0.24)	3,160	(53.25)	138	(2.33)	30	(0.51)	3	(0.05)	173	(2.92)	47	(0.79)	188	(3.17)	30	(0.51)	53	(0.89)	178	(3.00)	110	(1.85)	1,639	(27.62)

### Seasonality

*Shigella* isolates were recovered routinely throughout the study but were frequently recovered in the summer months (June to September; t = 7.83, *p* < 0.001; Table 2). Isolation of *Shigella* almost always peaked in July and August; 2003 and 2008 were exceptional years during which a September peak was observed. As indicated in the Beijing weather information, the temperature in September 2003 reached 33.7°C, the highest on record for the past 42 years (Provided by China Meteorological Data Sharing Service System, http://cdc.cma.gov.cn/home.do,). In September 2008, the amount of rainfall was 98.1 mm, which was twice the amount of rainfall during the same month in 2007 (Provided by China Meteorological Data Sharing Service System, http://cdc.cma.gov.cn/home.do). Both factors may have contributed to the late seasonal peaks.

**Table 2:** **Distribution of *Shigella *spp. by Months in Beijing from 1994 to 2010**

**Surveillance time**	**No. (%) of isolates**																									
	**Total**		**Jan**		**Feb**		**Mar**		**Apr**		**May**		**Jun**		**Jul**		**Aug**		**Sep**		**Oct**		**Nov**		**Dec**	
1994	620	(10.34)	5	(0.81)	3	(0.48)	3	(0.48)	2	(0.32)	12	(1.94)	56	(9.03)	165	(26.61)	178	(28.71)	122	(19.68)	42	(6.77)	23	(3.71)	9	(1.45)
1995	793	(13.22)	10	(1.26)	3	(0.38)	11	(1.39)	16	(2.02)	49	(6.18)	132	(16.65)	239	(30.14)	166	(20.93)	76	(9.58)	46	(5.80)	38	(4.79)	7	(0.88)
1996	1196	(19.94)	25	(2.09)	12	(1.00)	9	(0.75)	5	(0.42)	45	(3.76)	125	(10.45)	376	(31.44)	317	(26.51)	185	(15.47)	61	(5.10)	16	(1.34)	20	(1.67)
1997	481	(8.02)	15	(3.12)	5	(1.04)	11	(2.29)	19	(3.95)	55	(11.43)	76	(15.80)	85	(17.67)	66	(13.72)	68	(14.14)	52	(10.81)	15	(3.12)	14	(2.91)
1998	602	(10.04)	4	(0.66)	5	(0.83)	4	(0.66)	10	(1.66)	39	(6.48)	82	(13.62)	108	(17.94)	136	(22.59)	94	(15.61)	83	(13.79)	24	(3.99)	13	(2.16)
1999	477	(7.95)	3	(0.63)	5	(1.05)	3	(0.63)	7	(1.47)	17	(3.56)	63	(13.21)	109	(22.85)	129	(27.04)	80	(16.77)	29	(6.08)	26	(5.45)	6	(1.26)
2000	437	(7.28)	4	(0.92)	2	(0.46)	2	(0.46)	2	(0.46)	11	(2.52)	44	(10.07)	101	(23.11)	154	(35.24)	85	(19.45)	29	(6.64)	0	(0.00)	3	(0.69)
2001	221	(3.68)	0	(0.00)	1	(0.45)	2	(0.90)	1	(0.45)	1	(0.45)	8	(3.62)	41	(18.55)	139	(62.90)	26	(11.76)	2	(0.90)	0	(0.00)	0	(0.00)
2002	399	(6.65)	8	(2.01)	5	(1.25)	17	(4.26)	20	(5.01)	19	(4.76)	49	(12.28)	82	(20.55)	88	(22.06)	65	(16.29)	26	(6.52)	13	(3.26)	7	(1.75)
2003	94	(1.57)	0	(0.00)	1	(1.06)	2	(2.13)	0	(0.00)	1	(1.06)	5	(5.32)	15	(15.96)	24	(25.53)	29	(30.85)	13	(13.83)	2	(2.13)	2	(2.13)
2004	252	(4.20)	1	(0.40)	0	(0.00)	0	(0.00)	0	(0.00)	5	(1.98)	12	(4.76)	56	(22.22)	102	(40.48)	48	(19.05)	18	(7.14)	5	(1.98)	5	(1.98)
2005	148	(2.47)	4	(2.70)	1	(0.68)	3	(2.03)	0	(0.00)	0	(0.00)	1	(0.68)	24	(16.22)	53	(35.81)	39	(26.35)	15	(10.14)	6	(4.05)	2	(1.35)
2006	117	(1.95)	0	(0.00)	0	(0.00)	2	(1.71)	0	(0.00)	4	(3.42)	2	(1.71)	21	(17.95)	41	(35.04)	28	(23.93)	17	(14.53)	2	(1.71)	0	(0.00)
2007	78	(1.30)	1	(1.28)	2	(2.56)	0	(0.00)	1	(1.28)	1	(1.28)	3	(3.85)	17	(21.79)	38	(48.72)	10	(12.82)	3	(3.85)	0	(0.00)	2	(2.56)
2008	22	(0.37)	1	(4.55)	0	(0.00)	0	(0.00)	1	(4.55)	0	(0.00)	1	(4.55)	1	(4.55)	6	(27.27)	12	(54.55)	0	(0.00)	0	(0.00)	0	(0.00)
2009	36	(0.60)	0	(0.00)	0	(0.00)	2	(5.56)	6	(16.67)	0	(0.00)	1	(2.78)	1	(2.78)	16	(44.44)	7	(19.44)	2	(5.56)	0	(0.00)	1	(2.78)
2010	26	(0.43)	1	(3.85)	1	(3.85)	1	(3.85)	1	(3.85)	1	(3.85)	6	(23.08)	1	(3.85)	7	(26.92)	4	(15.38)	2	(7.69)	0	(0.00)	1	(3.85)
Total	5,999	(100.00)	82	(1.37)	46	(0.77)	72	(1.20)	91	(1.52)	260	(4.33)	666	(11.10)	1442	(24.04)	1660	(27.67)	978	(16.30)	440	(7.33)	170	(2.83)	92	(1.53)

### Age distribution

Epidemiological information was available for all 5999 patients. Patient age ranged from 3 months to 90 years. The age distribution for the 5934 cases of *S. flexneri* and S. *sonnei* is shown in Table 3. Adults aged between 21 and 25 years were the most commonly affected group (n = 978; 16.3%), followed closely by children aged less than 6 years (n = 821; 13.6%). *S. flexneri* 2a and *S. sonnei* were recovered from patients in each age group, although most infections caused by *S. sonnei* were found in children (n = 1639; 48%); children aged 0 to 5, 6 to 10, and 11 to 15 years accounted for 17.6% (n = 289), 15.2% (n = 250), and 14.8% (n = 242) of the cases, respectively. *S. flexneri* 2a occurred frequently in adults, especially those in the 21 to 25 (n = 594; 18.8%) and 26 to 30 (n = 380, 12%) age groups, although a high percentage (n = 400; 12.7%) was found to affect children aged less than 6 years.

**Table 3:** **Distribution of *S. flexneri *and *S. sonnei serotypes *among patients with shigellosis in Beijing between 1994 and 2010 (by age group)**

**Age groups**	**No. (%) of isolates in serotypes**																													
	**Total**		***S. flexneri *serotype 1a**		***S. flexneri *serotype 1b**		***S. flexneri *serotype 2a**		***S. flexneri *serotype 2b**		***S. flexneri *serotype 3a**		***S. flexneri *serotype 3b**		***S. flexneri *serotype 4a**		***S. flexneri *serotype 4b**		***S. flexneri *serotype 4c**		***S. flexneri *serotype 5**		***S. flexneri *serotype 6**		***S. flexneri *serotype x**		***S. flexneri *serotype y**		***S. sonnei***	
0–2	358	(6.03)	8	(4.68)	0	(0.00)	198	(6.28)	3	(2.17)	2	(6.67)	1	(33.33)	12	(6.94)	3	(6.38)	0	(0.00)	0	(0.00)	5	(9.43)	18	(10.11)	9	(8.18)	99	(6.04)
2–5	463	(7.80)	10	(5.85)	2	(14.29)	202	(6.41)	9	(6.52)	1	(3.33)	1	(33.33)	19	(10.98)	0	(0.00)	5	(2.66)	2	(6.67)	4	(7.55)	13	(7.30)	5	(4.55)	190	(11.59)
6–10	667	(11.24)	16	(9.36)	3	(21.43)	307	(9.74)	10	(7.25)	5	(16.67)	0	(0.00)	23	(13.29)	3	(6.38)	18	(9.57)	5	(16.67)	1	(1.89)	18	(10.11)	8	(7.27)	250	(15.25)
11–15	549	(9.25)	12	(7.02)	0	(0.00)	232	(7.36)	13	(9.42)	0	(0.00)	0	(0.00)	20	(11.56)	4	(8.51)	15	(7.98)	2	(6.67)	4	(7.55)	3	(1.69)	2	(1.82)	242	(14.77)
16–20	568	(9.57)	17	(9.94)	1	(7.14)	275	(8.72)	16	(11.59)	5	(16.67)	0	(0.00)	14	(8.09)	15	(31.91)	13	(6.91)	2	(6.67)	2	(3.77)	11	(6.18)	19	(17.27)	178	(10.86)
21–25	979	(16.50)	37	(21.64)	3	(21.43)	601	(18.84)	31	(22.46)	7	(23.33)	1	(33.33)	34	(19.65)	5	(10.64)	57	(30.32)	1	(3.33)	9	(16.98)	44	(24.72)	26	(23.64)	123	(7.50)
26–30	600	(10.11)	19	(11.11)	0	(0.00)	380	(12.05)	17	(12.32)	3	(10.00)	0	(0.00)	11	(6.36)	5	(10.64)	21	(11.17)	2	(6.67)	10	(18.87)	21	(11.80)	17	(15.45)	94	(5.74)
31–35	420	(7.08)	18	(10.53)	1	(7.14)	281	(8.91)	11	(7.97)	2	(6.67)	0	(0.00)	9	(5.20)	3	(6.38)	12	(6.38)	1	(3.33)	7	(13.21)	12	(6.74)	6	(5.45)	57	(3.48)
36–40	476	(8.02)	8	(4.68)	2	(14.29)	163	(5.17)	12	(8.70)	3	(10.00)	0	(0.00)	5	(2.89)	4	(8.51)	10	(5.32)	1	(36.67)	2	(3.77)	14	(7.87)	2	(1.82)	240	(14.64)
41–45	195	(3.29)	7	(4.09)	0	(0.00)	130	(4.12)	3	(2.17)	0	(0.00)	0	(0.00)	5	(2.89)	0	(0.00)	12	(6.38)	1	(3.33)	2	(3.77)	8	(4.49)	1	(0.91)	26	(1.59)
46–50	126	(2.12)	4	(2.34)	1	(7.14)	68	(2.16)	3	(2.17)	0	(0.00)	0	(0.00)	9	(5.20)	1	(2.13)	9	(4.79)	1	(3.33)	1	(1.89)	1	(0.56)	0	(0.00)	28	(1.71)
51–55	116	(1.95)	3	(1.75)	1	(7.14)	69	(2.19)	3	(2.17)	0	(0.00)	0	(0.00)	5	(2.89)	2	(4.26)	7	(3.72)	0	(0.00)	2	(3.77)	4	(2.25)	5	(4.55)	15	(0.92)
56–60	100	(1.69)	2	(1.17)	0	(0.00)	63	(2.00)	1	(0.72)	0	(0.00)	0	(0.00)	2	(1.16)	1	(2.13)	2	(1.06)	0	(0.00)	1	(1.89)	6	(3.37)	2	(1.82)	20	(1.22)
61–65	123	(2.07)	3	(1.75)	0	(0.00)	78	(2.47)	1	(0.72)	0	(0.00)	0	(0.00)	1	(0.58)	0	(0.00)	3	(1.60)	1	(3.33)	1	(1.89)	0	(0.00)	3	(2.73)	32	(1.95)
66–70	89	(1.50)	1	(0.58)	0	(0.00)	53	(1.68)	2	(1.45)	0	(0.00)	0	(0.00)	3	(1.73)	1	(2.13)	2	(1.06)	0	(0.00)	1	(1.89)	2	(1.12)	3	(2.73)	21	(1.28)
71–75	67	(1.13)	5	(2.92)	0	(0.00)	43	(1.36)	3	(2.17)	2	(6.67)	0	(0.00)	0	(0.00)	0	(0.00)	0	(0.00)	1	(3.33)	0	(0.00)	1	(0.56)	0	(0.00)	12	(0.73)
76–80	25	(0.42)	1	(0.58)	0	(0.00)	11	(0.35)	0	(0.00)	0	(0.00)	0	(0.00)	1	(0.58)	0	(0.00)	2	(1.06)	0	(0.00)	1	(1.89)	1	(0.56)	2	(1.82)	6	(0.37)
81–85	10	(0.17)	0	(0.00)	0	(0.00)	5	(0.16)	0	(0.00)	0	(0.00)	0	(0.00)	0	(0.00)	0	(0.00)	0	(0.00)	0	(0.00)	0	(0.00)	1	(0.56)	0	(0.00)	4	(0.24)
86–90	3	(0.05)	0	(0.00)	0	(0.00)	1	(0.03)	0	(0.00)	0	(0.00)	0	(0.00)	0	(0.00)	0	(0.00)	0	(0.00)	0	(0.00)	0	(0.00)	0	(0.00)	0	(0.00)	2	(0.12)
Total	5,934	(100.00)	17	(2.88)	1	(0.24)	3,160	(53.25)	13	(2.33)	3	(0.51)	3	(0.05)	17	(2.92)	47	(0.79)	188	(3.17)	3	(0.51)	53	(0.89)	178	(3.00)	110	(1.85)	1,639	(27.62)

### Gender

Information about patient gender was known for all of the patients infected with *S. flexneri* and *S. sonnei*; distribution was slightly biased toward male patients (n = 3616; 60%; Table 4). Unexpectedly, the ratio for some of the predominant serotypes of *S. flexneri* and *S. sonnei* differed in distribution between male and female patients. The most prevalent *S. flexneri* serotype (2a) was found more frequently in males (63.5%, *p* < 0.0001) than in females (36.5%). This result suggests that in Beijing, males either have greater exposure or are more susceptible to this subserotype than females. Similarly, *S. flexneri* 5 affected more males than females (ratio of infected males to females, 28 [46%]:5 [44%]; *p* < 0.001), although this serotype is not common in Beijing. By contrast, *S. flexneri* 2b and 6 as well as *S. sonnei* were more often associated with women than with men (*p* < 0.003, *p* < 0.04, and *p* < 0.0001, respectively).

**Table 4:** Distribution of the serotypes in Beijing from 1994 to 2010 (by gender)

***Shigella *species and serotypes**	**No. (%) of isolates**				**Total**	**Chi-square test**
	**Female**		**Male**			**p value**
*S*. *flexneri* serotype 1a	59	(0.99)	112	(1.89)	171	p < 0.03
*S*. *flexneri* serotype 1b	*6*	*(0.10)*	*8*	*(0.13)*	*14*	*p > 0.1*
*S*. *flexneri* serotype 2a	1,152	(19.41)	2008	(33.84)	3,160	p < 0.001
*S*. *flexneri* serotype 2b	71	(1.20)	67	(1.13)	138	p < 0.003
*S*. *flexneri* serotype 3a	8	(0.13)	22	(0.37)	30	p > 0.05
*S*. *flexneri* serotype 3b	2	(0.03)	1	(0.02)	3	p > 0.1
*S*. *flexneri* serotype 4a	71	(1.20)	102	(1.72)	173	p > 0.05
*S*. *flexneri* serotype 4b	14	(0.24)	33	(0.56)	47	p > 0.05
*S*. *flexneri* serotype 4c	75	(1.26)	113	(1.90)	188	p > 0.05
*S*. *flexneri* serotype 5	3	(0.05)	27	(0.46)	30	p < 0.001
*S*. *flexneri* serotype 6	29	(0.49)	24	(0.40)	53	p < 0.04
*S*. *flexneri* serotype x	68	(1.15)	110	(1.85)	178	p > 0.05
*S*. *flexneri* serotype y	40	(0.67)	70	(1.18)	110	p > 0.05
*S*. *sonnei*	719	(12.12)	920	(15.50)	1,639	p < 0.0001
Total	2,317	(39.00)	3,617	(61.00)	5,934	

## Discussion

According to a multicenter shigellosis surveillance study that incorporated data from six Asian sites, including China, shigellosis incidence is approximately 100-fold higher in Asia than in industrialized countries [5]. Other reports in China have indicated that shigellosis is even more ubiquitous than previously thought [18,19]. These studies have generally selected underdeveloped areas as surveillance sites, with little current information available on the epidemiologic trends in large cities like Beijing.

To begin to address this knowledge gap, the present study tracked *Shigella* infections in the largest infectious hospital in Beijing over a period of 17 years (1994 to 2010), thus providing a picture of serotype distribution across seasons, ages, and gender. The changing trend of *Shigella* in Beijing over time was determined in this work. The results will be useful in providing information that can be used by policy makers to implement control strategies or to predict the efficacy of vaccines for the prevention of shigellosis.

This study also demonstrated the diversity of all four species in recent years; the overall numbers of *S. flexneri* and *S. sonnei* have decreased, whereas the numbers of *S. dysenteriae* and *S. boydii* have not. *S. flexneri* 2a*,* the dominant serotype since 1994, was found to have been overtaken by *S. sonnei* in 2000; however, *S. flexneri* remained the dominant species in Beijing until 2006. The prevailing *Shigella* spp. in developing countries is *S. flexneri,* whereas that in developed countries is *S. sonnei*[14]. The same transition of dominant *Shigella* species observed not only in Beijing but also in southern Vietnam [20] and in Dhaka [21], all of which have undergone rapid economic development in recent years; the phenomenon supporting the circulating species and serotypes may be considered as a marker of the socioeconomic climate in an individual setting [22]. This gross economic improvement may have positively affected the quality of drinking water and the overall behavioral change in personal hygiene. These changes in turn may have influenced host characteristics, including the reduction of overall malnutrition that leads to an improved immune response. The genetic mutation of non-virulent microbes into pathogenic ones [23] may have also played an important role in the emergence of *S. sonnei*.

In this study, the 2006 data were compared with that of another similar report based on a study in Henan Province, China, in 2006 [18]. *Shigella* infection in Beijing reached its peak in August 2006, whereas that in Henan Province reached its peak in July 2006. For the same period, the data from the China Meteorological Administration (http://cdc.cma.gov.cn/home.do) revealed that the highest temperature in Beijing was recorded in August, whereas that in Zhengzhou, Henan Province, was recorded in July. Rainfall for this year peaked in July for both provinces. The high temperatures appear to more related to the seasonality of the *Shigella* infection than to the amount of rainfall; the same has also been observed in southern Vietnam [20].

Interestingly, the top three most common *S. flexneri* serotypes from 1994 to 2010 were *S. flexneri* 2a*,* 4c, and x; overall, *S. flexneri* 2a ranked first, but *S. flexneri* 4c was the most dominant *S. flexneri* in 2004 and 2005*.* The same result was reported in Henan Province in 2006, during which *S. flexneri* 4c was the most frequently isolated serotype [18]. The distribution of *S. flexneri* serotypes between Beijing and other areas in China was highly heterogeneous from site to site and year to year. The observation that *S. flexneri* 4c had become dominant in Beijing and Henan suggests the spread of *S. flexneri* 4c throughout China*.* This serotype was first isolated in the USSR in the 1980s [24]. Similar to Beijing*, S. flexneri* 4c became the most predominant serotype in Zhejiang Province [25]. The factors contributing to the rise and the fall of *S. flexneri* 4c in China have not yet been fully explored. This study also highlighted the observation that age influenced the serotype distribution within the *Shigella* spp. Skewed gender distribution toward males was found for *S. sonnei* infection, which affected more males than females in Beijing; the same was also observed in Henan Province, China [18].

In conclusion, the change across 17 years in dominant *Shigella* species from *S. flexneri* to *S. sonnei* was uncovered. The results would be important in the treatment and prevention of shigellosis or in the implementation of vaccine strategies. The results also highlights the need for continuous monitoring of *Shigella* in the future.

### Consent

Written informed consent was obtained from the patient for the publication of this report and any accompanying images.

## Competing interests

The authors declared that they have no competing interests.

## Authors’ contributions

YM, EC, CB, ZL, SC, JZ, HW, CZ performed the experiments and provided clinical samples and patient data, JZ analyzed the data, JDK and BZ wrote the first draft of the manuscript, FQ and ZW designed the study, supervised the experiments and contributed to the manuscript. All authors read and approved the final manuscript.
